# Network‐based rTMS to modulate working memory: The difficult choice of effective parameters for online interventions

**DOI:** 10.1002/brb3.2361

**Published:** 2021-10-15

**Authors:** Lysianne Beynel, Moritz Dannhauer, Hannah Palmer, Susan A. Hilbig, Courtney A. Crowell, Joyce E‐H. Wang, Andrew M. Michael, Eleanor A. Wood, Bruce Luber, Sarah H. Lisanby, Angel V. Peterchev, Roberto Cabeza, Simon W. Davis, Lawrence G. Appelbaum

**Affiliations:** ^1^ Department of Psychiatry and Behavioral Science Duke University School of Medicine Durham North Carolina USA; ^2^ Duke Institute for Brain Sciences Duke University School of Medicine Durham North Carolina USA; ^3^ Noninvasive Neuromodulation Unit Experimental Therapeutics and Pathophysiology Branch National Institute of Mental Health Bethesda Maryland USA; ^4^ Department of Biomedical Engineering Duke University School of Medicine Durham North Carolina USA; ^5^ Department of Electrical and Computer Engineering Duke University School of Medicine Durham North Carolina USA; ^6^ Department of Neurosurgery Duke University School of Medicine Durham North Carolina USA; ^7^ Center for Cognitive Neuroscience Duke University Durham North Carolina USA; ^8^ Department of Psychology & Neuroscience Duke University Durham North Carolina USA; ^9^ Department of Neurology Duke University School of Medicine Durham North Carolina USA

**Keywords:** DLPFC, parietal cortex, repetitive transcranial magnetic stimulation, working memory

## Abstract

**Background:**

Online repetitive transcranialmagnetic stimulation (rTMS) has been shown to modulate working memory (WM) performance in a site‐specific manner, with behavioral improvements due to stimulation of the dorsolateral prefrontal cortex (DLPFC), and impairment from stimulation to the lateral parietal cortex (LPC). Neurobehavioral studies have demonstrated that subprocesses of WM allowing for the maintenance and manipulation of information in the mind involve unique cortical networks. Despite promising evidence of modulatory effects of rTMS on WM, no studies have yet demonstrated distinct modulatory control of these two subprocesses. The current study therefore sought to explore this possibility through site‐specific stimulation during an online task invoking both skills.

**Methods:**

Twenty‐nine subjects completed a 4‐day protocol, in which active or sham 5Hz rTMS was applied over the DLPFC and LPC in separate blocks of trials while participants performed tasks that required either maintenance alone, or both maintenance and manipulation (alphabetization) of information. Stimulation targets were defined individually based on fMRI activation and structural network properties. Stimulation amplitude was adjusted using electric field modeling to equate induced current in the target region across participants.

**Results:**

Despite the use of advanced techniques, no significant differences or interactions between active and sham stimulation were found. Exploratory analyses testing stimulation amplitude, fMRI activation, and modal controllability showed nonsignificant but interesting trends with rTMS effects.

**Conclusion:**

While this study did not reveal any significant behavioral changes in WM, the results may point to parameters that contribute to positive effects, such as stimulation amplitude and functional activation.

## Graphical abstract

Working memory is a crucial a cognitive ability that declines rapidly with aging. Repetitive transcranial magnetic stimulation (rTMS) has been proposed as an approach to enhance working memory abilities, however effects are often modest. In this study, advanced techniques were used (fMRI, network controllability and electric field modeling) to optimize rTMS effects. However, no significant differences were found between active and sham rTMS, as such the manuscript provides a detailed account of how stimulation and task parameters may have led to the lack of significant rTMS results.

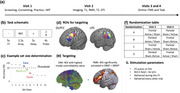

## 1 INTRODUCTION

Working memory (WM) is an essential cognitive ability that is central to many aspects of daily living (Barrett et al., 2004). While many studies are seeking to affect WM performance through the use of repetitive transcranial magnetic stimulation (rTMS) (Koch et al., 2005; Luber et al., 2007; Mottaghy et al., 2003), meta‐analytic reviews of these studies suggest that reliable technique for inducing offline (Patel et al., 2020) and online performance enhancement have not yet emerged (Beynel, Appelbaum et al., 2019). As such, there is a need to test novel targeting schemes that incorporate multiple forms of cognitive, neuroanatomical, and neurophysiological information to optimize neuromodulation‐based WM enhancement.

fMRI studies have demonstrated that the fronto‐parietal network is a main driver of WM performance with activations of both the dorsolateral prefrontal cortex (DLPFC) and the parietal cortex (e.g., see Emch et al., 2019 for a meta‐analysis). Therefore, these two loci are often targeted in noninvasive brain stimulation studies such as rTMS and transcranial electrical stimulation studies, with results showing significant effects, but often modest effect sizes (see Brunoni & Vanderhasselt, 2014 for a review). One of the potential ways to improve brain stimulation efficacy on WM is to use *online* rTMS, which is applied as participants perform the WM task, under the hypothesis that this will lead to greater Hebbian‐like plasticity.

In two previous studies (Beynel, Appelbaum et al., 2019; Beynel, Davis et al., 2020) our team has used online rTMS in healthy younger and older adults, as a means to improve WM and mitigate cognitive decline. In these studies, rTMS was applied while participants performed a delayed‐response alphabetization task (DRAT), in which they were asked to maintain in WM and to reorder by alphabetical order, an array of letters. After a delay, participants were cued to respond to a letter and number combination. Their task was to report if the probe letter appeared in the original array and if the number matched the serial position of the letter in the reorganized alphabetical order (“Valid”), if the letter was in the original array but the number did not match the serial position in the alphabetized set (“Invalid”), or if the letter was not in the original array (“New”). Given the importance of the fronto‐parietal network in supporting WM maintenance and manipulation (Rottschy et al., 2012; Wager & Smith, 2003), rTMS was applied to either the left DLPFC (Beynel, Davis et al., 2019) or the left lateral parietal cortex (LPC, Beynel, Davis et al., 2020) using individualized fMRI‐guided targeting. Since WM has been shown to rely on coactivation between these brain regions occurring in the theta (approximately 5 Hz) frequency range (Berger et al., 2019; Riddle et al., 2020), and since rTMS can entrain brain oscillations at this frequency (Thut et al., 2011), rTMS was applied at 5 Hz. Results from these studies demonstrated a pattern of site‐specific effects of rTMS with performance enhancement when stimulation was targeted at DLPFC and performance disruption when LPC was stimulated. However, for both of these studies the superiority of active rTMS over sham was limited. Exploratory analyses revealed that both fMRI activation and modal controllability—defined with diffusion weighted imaging (DWI) as the ability of a node to steer the brain into a hard‐to‐reach state—significantly predicted the magnitude of rTMS effects on WM performance (Beynel, Deng et al., 2020).

In the current study, to build on these previous findings and to obtain stronger rTMS effects, we implemented, for the first time, three neuroimaging‐based targeting parameters: modal controllability combined with fMRI activation to define the rTMS targets; and E‐field modeling to define rTMS amplitude using an approach developed by our team (Beynel, Davis et al., 2020). Finally, to test different elements of memory processing that may underlie WM, a delayed‐response maintenance task (DRMT) in which no alphabetical reordering was required, was also performed.

While this study builds on previous experiments from our group, all data presented in this manuscript are original data. Based on these previous studies, we expected to find opposite rTMS effects during the DRAT with performance enhancement when applied over the DLPFC and disruption when applied over the LPC. Since WM maintenance has been more closely associated with parietal cortex function (Hamidi et al., 2008; Luber et al., 2007), while manipulation of items in WM is reported to involve frontal cortex (Postle, 2006), we expected to find performance enhancement on the DRMT for rTMS applied over the LPC only. As such, this study was designed to expand the state‐of‐the‐art by employing multiple cortical targets, distinct WM task demands, and equivalency dosing of rTMS, in a repeated measures design within the same experimental protocol.

## 2 METHODS

### 2.1 Participants

Fifty‐nine participants (18–35 years old) were recruited to participate in this single‐blind, randomized, within‐subject, placebo‐controlled study, approved by the Duke University School of Medicine Institutional Review Board (IRB protocol #Pro00065334), and preregistered on ClinicalTrials.gov (NCT02767323). Participants were excluded during the first visit if they had contraindications to Magnetic Resonance Imaging (MRI) (*n* = 4), or Transcranial magnetic stimulation (TMS) (*n* = 8), experienced syncope during motor threshold assessment (*n* = 1), or demonstrated poor behavioral performance, as reflected by the sigmoid function, during initial practice with the task (*n* = 2). The remaining 44 participants were asked to come back for three subsequent visits including an imaging visit and two TMS visits. Of these individuals, one was excluded because of poor task performance, five withdrew because of scheduling conflicts, and seven withdrew during the TMS visits because of TMS‐induced pain (*n* = 4), headache (*n* = 1), or anxiety (*n* = 1), with no (or only partial) data collected from these subjects. Finally, because of the Covid‐19 pandemic, the study protocol was suspended and participation of three participants was terminated. Twenty‐nine healthy young adults completed the full protocol (mean age: 23.9 ± 4.3 years old; 16 females and 13 males). All participants had normal or corrected‐to‐normal visual acuity, were native English‐speakers, and were compensated $20 per hour for their efforts with a $100 completion bonus at the end of all study activities.

### 2.2 Experimental protocol

**FIGURE 1:**
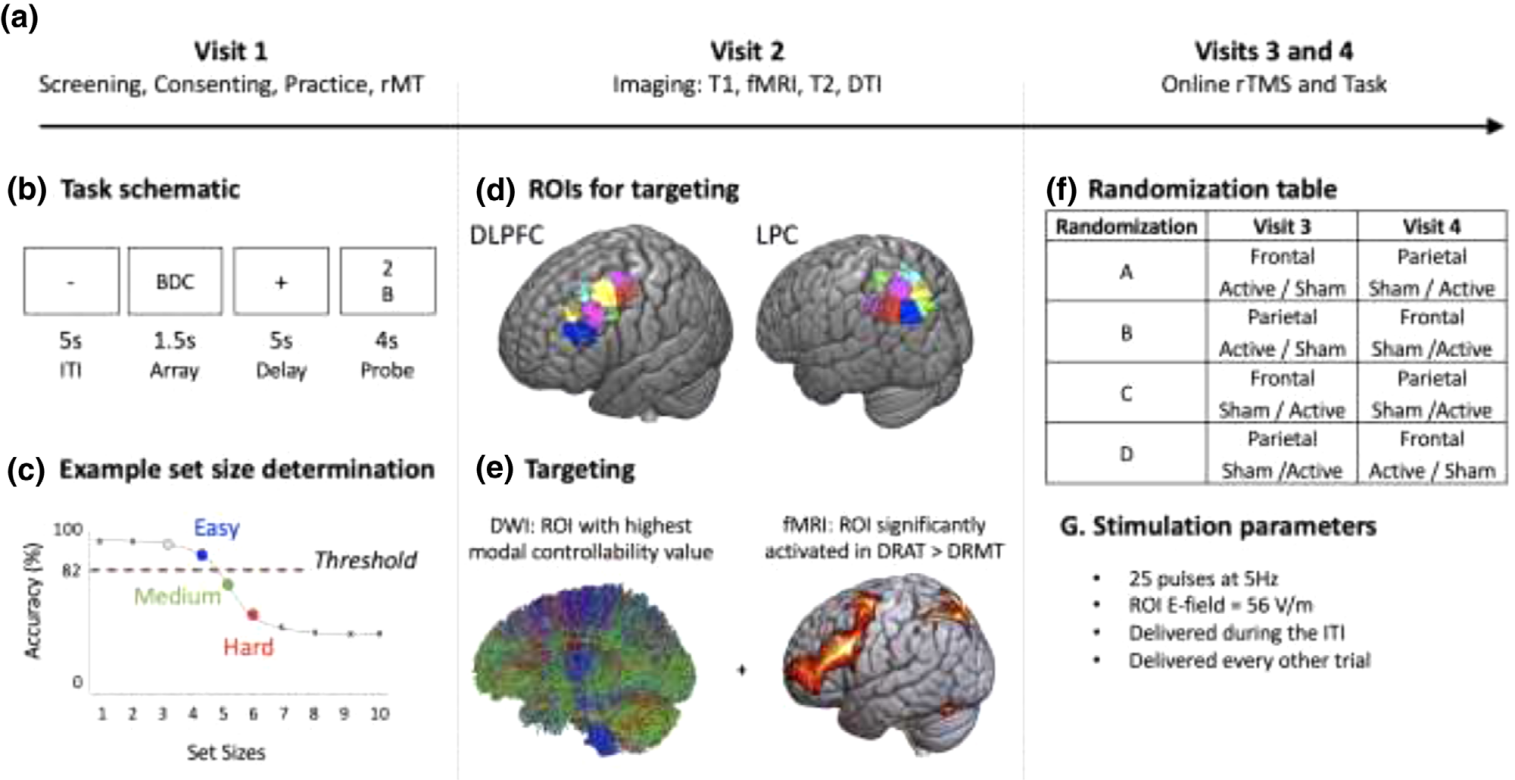
(a) Study visits. (b) Task schematic of a single trial (c) Set size determination using sigmoidal fit (d) Illustration of the nine regions of interest (ROIs) within the DLPFC and eight ROIs within the LPC that were used as potential TMS targets (colors represent different ROIs from the Harvard Oxford Atlas). (e) Illustration of the targeting approach combining DWI and fMRI. (f) Randomization table used to define stimulation target and type on each TMS visits. (g). rTMS parameters

As illustrated in Figure 1a, this study involved four visits. The first visit included screening, consenting, task practice, and resting motor threshold (rMT) determination. The second visit, which occurred on average 18 days after the first visit, was done at the Duke‐UNC Brain and Imaging Analysis Center (BIAC) and included structural and functional neuroimaging. Online rTMS during the WM tasks was performed on the third and fourth visits, which occurred on average 16 days after the second visit and within, on average, five days apart of each other. Information about each visit is provided below and more details can be found in Beynel, Davis et al. (2020) and Beynel, Davis et al. (2019)

#### 2.2.1 Visit 1: Consenting, screening, rMT, task practice, and set size determination

During the first visit, written informed consent was obtained, followed by inclusion and exclusion screening to ensure participants did not have contra‐indication to MRI or TMS, as defined the TMS adult safety screen (Keel et al., 2001). The MINI‐International Psychiatric Interview (Sheehan et al., 1998) was assessed to ensure participants did not have current or past psychiatric disorders. All participants were also screened for substance use with urine drug tests and women were screened with urine pregnancy tests.

Participants who did not meet inclusion criteria were thanked, compensated for their time, and left the study. All other participants underwent a resting motor threshold (rMT) determination procedure. For this purpose, TMS was performed using a figure‐of‐8 coil (A/P Cool‐B65) and a MagProX100 stimulator (Magventure Denmark), set up to deliver biphasic pulses in the standard pulse mode current direction (AP/PA). Electrodes (Neuroline 720, Ambu, USA) were placed in a belly‐tendon montage over the right first dorsal interosseous (FDI). Motor evoked potentials (MEP) were digitized and recorded on a BrainSight neuronavigation system (Rogue Research, Canada) that saved the position of the coil on the participant's head, registered on a template MNI brain, for each TMS pulse. The hotspot was defined as the optimal location eliciting the largest MEP in the FDI and rMT was determined using a maximum likelihood estimation procedure (TMS Motor Threshold Assessment Tool, MTAT 2.0 (Awiszus, 2003). While the individual rMT was not used to define the rTMS amplitude, since this was selected based on computational E‐field modeling as described below, rMT was used to ensure that the stimulation amplitude would not exceed safety guidelines (Rossi et al., 2009). To conclude the first visit, participants were asked to learn and practice the two WM tasks, performing six blocks of the DRAT, followed by two blocks of the DRMT.

In each task (illustrated schematically in Figure 1b), the trial began with an intertrial interval (ITI) of 5 s, followed by presentation of an array of letters that was shown for 1.5 s then replaced by fixation cross for 5 s, and concluded with display of a letter/number probe for 4 s. During the DRAT blocks, participants were asked to reorder the letters alphabetically during the delay and respond to the probe as Valid if the number matched the serial position of the letter in the alphabetized list, and as Invalid if it did not match. In the DRMT task, participants responded Valid if the number matched the serial position of the probe letter in the original (unalphabetized) list, and Invalid if it did not. In both cases, participants were asked to press “1” if the number matches, or “2” if they did not, using a keyboard. Each block included 35 trials, with the set size varied based on performance on the previous trial according to a 2‐down‐1‐up staircase procedure.

Once complete, these practice data were used to establish individualized difficulty levels for the subsequent sessions by fitting the accuracy on the DRAT with a sigmoid function (Figure 1c). According to this procedure, difficulty levels were calculated for each participant relative to an 82% accuracy threshold (Beynel, Davis et al., 2020) with Easy trials defined as the smallest set size with accuracy higher than threshold and Medium and Hard as the two set sizes subsequently lower along the fitted curve. In order to focus on the most challenging condition which has been shown to produce the greatest effects in previous studies (Beynel, Davis et al., 2019; Beynel, Davis et al., 2020; Luber et al., 2007; Viggiano et al., 2008; Violante et al., 2017) only the hard condition was presented during the rTMS visits for the first 15 participants (*Cohort 1*). This decision to include only one set size, however, resulted in unexpectedly high accuracy levels (see Section 3.1.1); in attempt to address this ceiling effect, the subject‐specific easy set size condition was added (randomized trial‐to‐trial) for the remaining 14 participants (*Cohort 2*) in order to provide participants with a range of difficulties across conditions.

#### 2.2.2 Visit 2: MRI acquisition

During the second visit, participants were scanned on a 3‐T MRI gradient echo scanner (General Electric 3.0 Tesla Sigma Excite HD short bore), equipped with an eight‐channel head coil. During this session, an anatomical MRI was collected (3D‐T1‐weighted echo‐planar sequence, acquisition matrix = 512 mm^2,^ time repetition [TR] = 2304 ms, time echo [TE] = 3.2 ms, field of view [FOV] = 256 mm^2^, spacing between slices = 0.5 mm, 166 slices), followed by four runs of coplanar EPI functional images acquired using an inverse spiral sequence acquisition matrix = 128 mm^2^, TR = 2 s, TE = 30 ms, FOV = 256 mm^2^, spacing between slices = 2 mm); and by a diffusion‐weighted imaging (DWI) (acquisition matrix = 144 mm^2^, TR = 4660 ms, TE = 64.7 ms, FOV = 220, spacing between slices = 1.5 mm, *b*‐value = 3000 s/mm^2^, diffusion‐sensitizing directions = 90). During the four runs of functional acquisition, participants were asked to perform the DRAT and the DRMT in a block‐design fashion. In each run, participants alternatively performed six trials of the DRAT and six trials of DRMT. The combination of tasks was repeated three times within each run. The order of the tasks was counterbalanced within each block and run. Both tasks were performed at the “Medium” difficulty level as defined by the results from the sigmoid function.

Stimuli for the tasks were back projected onto a screen located at the foot of the MRI bed using an LCD projector. Subjects viewed the screen via a mirror system located in the head coil and the start of each run was electronically synchronized with the MRI acquisition computer. Overall accuracy was presented on the screen at the end of each run. Behavioral responses were recorded with a 4‐key fiber‐optic response box (Resonance Technology, Inc.). Scanner noise was reduced with ear plugs, and head motion was minimized with foam pads. When necessary, vision was corrected using MRI‐compatible lenses that matched the distance prescription used by the participant.

#### 2.2.3 Visits 3 and 4: Online rTMS

On each TMS visit, participants were asked to perform 12 blocks of the two tasks with 30 trials per block at the hard difficulty level (*Cohort 1*) or at the easy and hard difficulty levels (*Cohort 2*). DRAT and DRMT were alternatively presented on every other block, and feedback was provided on each trial as “correct” in green or “incorrect” in red, based on subject responses. Accuracy performance for a given block was provided following its completion. For each cohort, this led to a total of 720 trials, divided across 16 conditions for Cohort 1 (Active/Sham, Frontal/Parietal, DRAT/DRMT, trial with and without pulses) and 32 conditions for Cohort 2 (same conditions + Easy/Hard difficulty levels). This led to a total of 45 trials per condition for Cohort 1 and ∼ 22 trials for Cohort 2.

The two visits were divided into halves with a brief, self‐paced break between the sixth and seventh blocks to allow time for rest and for the experimenter to switch the coil from the active to the sham configuration, or vice versa. Participants were randomized into one of four groups that defined the order of stimulation type (active or sham) and stimulation target (frontal or parietal) delivered during the two halves of the two visits (see Figure 1f). TMS procedures performed on visits 3 and 4 used the same devices described above for the rMT procedure. Twenty‐five pulses of active or sham rTMS were applied at 5Hz, on every other trial right before presentation of the letter array. The intensity was selected to deliver a fixed E‐field strength (E_ref_) at the target region of interest (ROI) in the active condition (see Section 2.4.1 and Figure 1g). During each visit, the TMS coil position was continually monitored through a stereotaxic neuronavigation system (Brainsight, Rogue Research, Canada) and maintained at a high level of precision with robotic guidance using a Smart Move Robot (Advanced Neuro Technology, Netherlands).

Sham stimulation was applied using the same intensity setting but with the coil in placebo mode. A low amplitude current stimulator is built into this coil, and is connected to two electrodes (Ambu, 72020/K/C with wet gel) that are placed directly underneath the TMS coil on the subject's scalp approximately 1.5–2cm edge‐to‐edge. This equipment, therefore, produced similar clicking sounds and somatosensory sensation (via electrical stimulation) as in the active mode, but without a significant magnetic field reaching the brain (Smith & Peterchev, 2018). To keep participants blinded, they were told at the beginning of the experiment that they would receive two different types of stimulation during the study. To test the efficacy of the blinding without revealing that there was a sham condition, they were asked whether they thought rTMS affected their performance on a scale from −10 to +10, with negative scores indicating performance disruption, positive scores indicating performance enhancement, and zero indicating no changes.

### 2.3 Targeting approach using fMRI, DWI, and coil positioning

This study built upon the results from Beynel, Deng et al. (2020) which demonstrated that fMRI activation and modal controllability significantly predicted rTMS effects. Therefore, these two predictors were used to define the stimulation targets for both the frontal and parietal cortices. To define TMS targets within these areas, we overlaid the fronto‐parietal network obtained from the Power atlas (Power et al., 2011) and looked for corresponding ROIs defined using the Harvard and Oxford atlas (HOA) distributed with FSL (https://fsl.fmrib.ox.ac.uk/fsl/fslwiki/Atlases). Using this procedure, 15 and 10 ROIs were identified in the frontal and parietal cortices, respectively. Some of these optional ROIs were excluded due to their location (the most anterior ROIs would induce painful stimulation of forehead muscles and coil placement that blocked the subject's view of the experimental stimuli). From these options, nine ROIs within the left mid‐frontal gyrus, and eight ROIs in the left lateral‐parietal cortex, (Figure 1e) were selected as potential TMS targets. Then, for each subject, fMRI analysis was performed to select the ROI which was significantly more activated by the DRAT than the DRMT, while also showing the strongest modal controllability (see fMRI and DWI analyses section below) (Figure 1f). Two conditions needed to be satisfied: at least 25% of the voxels within the ROI need to be significantly activated to go to the next step. Then, across the remaining ROIs, only the one with the highest modal controllability value is selected. The selected frontal and parietal ROIs in MNI space were then registered back to individual native space, using the FMRIB's Linear Image Registration Tool (FLIRT); and entered into the neuronavigation system (Brainsight, Rogue Research, Canada). The center of these two ROIs were selected as the final DLPFC and LPC targets.

**FIGURE 2:**
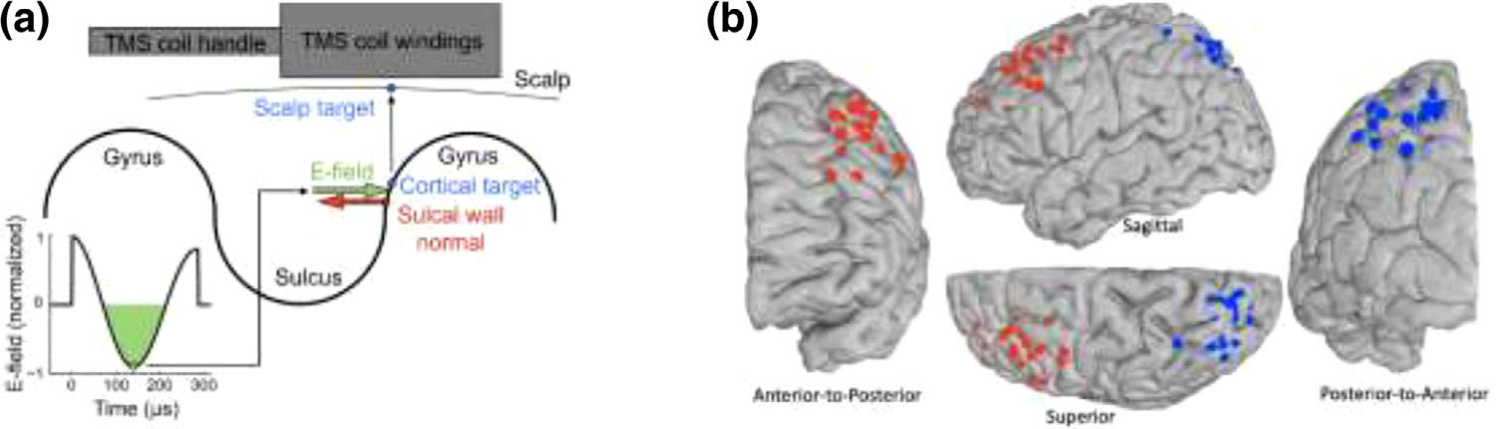
(a) Schematic illustrating E‐field modeling performed to obtain the coil orientation for each subject. (b) Locations of left DLPFC (red) and LPC (blue) targets for TMS. The brain surfaces of the experimental subjects as well as the Colin27 atlas were surface‐registered using Freesurfer MRI segmentation, as part of SimNIBS's mri2mesh pipeline, and used to visualize all individual TMS targets on the brain surface of the Colin27 atlas

To define the coil position and orientation for these two targets, their coordinates in native space were extracted and projected onto the scalp surface, using a nearest neighbor approach, and extended slightly outwards to account for the subject's hair thickness (Beynel, Davis et al., 2020). The compressed hair thickness was measured for each subject with a depth gauge (Digital Tread Depth Gauge, Audew, Hong Kong; resolution 0.01 mm) installed on a custom‐made plastic base placed over the target location at the beginning of each TMS visit. (The hair thickness measurement is critical mostly for the E‐field modeling described below.) The TMS coil was oriented around the scalp‐normal vector so that the direction of the second phase of the induced E‐field coincided with the inward‐pointing normal vector on the sulcal wall closest to the brain target location (Figure 2a). An angle representing the intended coil orientation was computed and entered in the neuronavigation system, using the “twist” tool. More details about this procedure can be found in the Supporting Information in Beynel et al. (2020).

The TMS target locations for the left frontal cortex and the left parietal cortex across subjects are depicted in Figure 2b. In more detail, a custom MATLAB script was first used to convert each TMS target locations in the native MRI to the FreeSurfer space by moving the coordinate origin into the volumetric center of the SimNIBS‐preprocessed MRI as well as flipping the *x* and *y* axis. The FreeSurfer space‐converted target coordinates were then projected to the nearest (minimal Euclidean distance, smaller than 1 mm for all subjects) brain surface (the FreeSurfer‐generated lh.pial file) node and used in further steps. During the head model creation in SimNIBS’ *mri2mesh* framework, the FreeSurfer software (version 5.3.0) established a surface‐based registration between the individuals brain surface and the FreeSurfer's average template model (i.e., through the *mris_register* command). The resulting FreeSurfer brain surface, for the relevant left hemisphere (i.e., the file lh.pial) was registered based on individual gyral/sulcal curvature information onto a spherical representation (i.e., stored as file lh.sphere.reg) of the template with a node‐to‐node correspondence. The SimNIBS pipeline was run on another head geometry, the MNI Colin27 atlas (Holmes et al., 1998), and its spherical FreeSurfer average template representation was used to convert and eventually visualize (through SCIRun 4.7 R45839) all individual TMS targets on Colin27's (i.e., lh.pial) brain surface as seen in Figure 2. More specifically, the identified locations on the subjects spherical representation of FreeSurfer's average template (i.e., lh.sphere.reg) were mapped to the closest node location of the one from Colin27 (i.e., lh.sphere.reg) in terms of minimal Euclidean distance (for all subjects < 1 mm), and then the brain surface (i.e., lh.pial) node with the corresponding index was plotted as a red or blue ball (of radius 2.5 mm) for DLPFC or LPC, respectively.

#### 2.3.1 fMRI and DWI analyses

Functional images were preprocessed using FMRIB's Software Library (FSL, https://fsl.fmrib.ox.ac.uk/fsl/fslwiki). Images were corrected for slice acquisition timing, motion, and linear trend; motion correction was performed using FSL's MCFLIRT, and six motion parameters estimated from the step were then regressed out of each functional voxel using standard linear regression. Images were then temporally smoothed with a high pass filter using a 190 s cutoff and normalized to the MNI stereotaxic space. Spatial filtering with a Gaussian kernel of FWHM of 6 mm was applied. A general linear model (GLM) was conducted, using the FEAT module of FSL, in which blocks of tasks were convolved as two different regressors with a double‐gamma hemodynamic response function. The *z*‐score map from the DRAT > DRMT contrast was obtained, and binarized to only display voxels with significant activation (*z* > 1.96, *p* < 0.05).

Information on the structural connections based on diffusion tractography between each pair of regions in our data was assessed with a standard DWI processing pipeline used previously in our group (Beynel, Davis et al., 2020; Davis et al., 2018; Davis et al., 2017). DWI data were analyzed using FSL and MRtrix (http://mrtrix.org) software packages. Data were denoised, corrected with eddy current correction, and bias‐field corrected using MRtrix (Tournier et al., 2012) and FSL. Constrained spherical deconvolution was used in calculating the fiber orientation distribution. After tracts were generated, they were filtered using spherical‐deconvolution informed filtering of tractograms (Smith et al., 2013). Tracts were SIFTed until 1 million tracts remained. Connectomes were then generated by using FLIRT to apply a linear registration to the Harvard Oxford Atlas (HOA) and register them to native diffusion space. Subsequent connectomes describe the number of streamlines connecting any pair of regions within this atlas, therefore resulting in a 471*471 matrix. The diagonal of this matrix was zeroed, and used to estimate modal controllability (https://github.com/skyeong/controllability) for all ROIs. The controllability values of the nine ROIs within the left DLPFC and the eight ROIs within the left LPC were extracted, and the two ROIs in the DLPFC and LPC that were significantly activated by the task while also showing the strongest modal controllability values (compared to the nine and eight other ROIs) were selected as the TMS targets.

### 2.4 rTMS amplitude individualization using electric field modeling

E‐field modeling was used to compute the coil current intensity (as a percentage of the maximum stimulator output, MSO) such that all subjects received TMS‐induced E‐field of the same strength (E_ref_) at the cortical target. To do that, we modeled the individual head, including hair thickness, and TMS coil setup (as described in Section 2.3 and Beynel, Davis et al., 2020) to simulate the individual E‐field distribution in the brain using SimNIBS (ver. 2.0.1; Thielscher et al., 2015). The computational model of each participant's head was generated using coregistered T1‐ and T2‐weighted MRI datasets to segment major tissue types (scalp, skull, cerebrospinal fluid, and gray and white matter) and represented them as tetrahedral mesh elements, which, besides gray and white matter being modeled anisotropically (derived from MRI‐DTI data), received literature‐based isotropic conductivity values. For the TMS coil setup, the coil center was placed at the scalp‐projected point of the ROI center and oriented perpendicular to the closest sulcus wall with the induced E‐Field pointing inwards. For each modeling setup, we calculated the E‐Field and evaluated E_100_ metric which represents the 100th largest E‐field magnitude across the voxels in the individual ROI as a robust measure of E‐Field exposure. Based on the simulation, we calculated the needed coil current intensity to make E_100_ match a reference value of *E*_ref_ = 56 V/m for any subject and ROI (DLPFC and LPC). This E_ref_ value was derived from population data relating the E‐field strength corresponding to TMS at rMT intensity in LPC and was found to be sufficient to induce significant behavioral change with active rTMS in LPC during the DRAT task used in our former study (Beynel, Davis et al., 2020). The equivalency between intensities as expressed by percentage of MSO and rMT are provided in Table 1 for each target.

**TABLE 1:** Resting motor threshold (rMT) and rTMS amplitude at dorsolateral prefrontal cortex (DLPFC) and lateral parietal cortex (LPC) TMS targets, each row represents one participant within each cohort, expressed both as a percentage of the resting motor threshold (rMT) and of the maximum stimulator output (MSO). Data are sorted by resting motor threshold amplitude (%MSO)

	rTMS pulse amplitude			
	DLPFC		LPC	
rMT (%MSO)	(% MSO)	(% rMT)	(% MSO)	(% rMT)
***Cohort 1***				
35	35	100	35	100
37	40	108	45	122
38	45	118	45	118
39	41	105	38	97
42	41	98	46	110
43	30	70	50	116
44	39	89	46	105
45	39	87	41	91
47	35	74	49	104
48	42	88	48	100
51	52	102	39	76
57	39	68	50	88
58	48	83	65	112
77	37	48	79	103
77	35	45	36	47
***Cohort 2***				
40	34	85	44	110
44	36	82	57	130
45	33	73	40	89
47	31	66	32	68
51	48	94	56	110
55	33	60	35	64
56	47	84	42	75
58	49	85	69	119
58	41	71	54	93
59	44	75	55	93
66	39	59	49	74
72	37	51	47	65
81	40	49	45	56
83	45	54	47	57
***Average across both cohorts***				
53.55	39.83	78.31	47.72	92.83

### 2.5 Planned statistical analyses

The central question of interest here is whether active rTMS led to differences in accuracy relative to sham and what factors modified such differences. Given the limited sample size, and the large number of factors: Condition (Valid and Invalid trials), Task (DRAT and DRMT), Target (DLPFC and LPC), Stimulation (Active and Sham rTMS), and Difficulty (Easy and Hard, for *Cohort 2*), a paired‐sample *t*‐test was first performed to assess the effect of Condition. Indeed, according to our former study (Beynel, Davis et al., 2020), no differences were expected between the two levels of this factor. Results from the *t*‐tests confirmed this assumption since no differences were found in *Cohort 1* (*t*_14_ = –1.05, *p* = 0.31) and in *Cohort 2* (*t*_13_ = −0.99, *p* = 0.34). Therefore, to reduce the number of factors, the data from Valid and Invalid trials were collapsed. Repeated measures ANOVAs (rANOVAs) were then performed with the four remaining factors of interest: Task (DRAT and DRMT), Target (DLPFC and LPC), Stimulation (Active and Sham rTMS), and Difficulty (Easy and Hard) only in *Cohort 2*. In all analyses, trials without TMS pulses and trials for which subjects did not answer were excluded before performing rANOVA. Analyses were performed with JASP (version 0.13.1, JASP Team, https://jasp-stats.org). As needed, post hoc analyses were performed with Bonferroni correction. Results are reported as mean ± standard deviation, with criterion alpha of *p* < 0.05. Effect sizes are reported with *η* ^2^ for significant results.

### 2.6 Exploratory statistical analyses

The present study involved multiple factors that may differentially contribute to the effects of rTMS on behavioral performance. In order to better understand how these factors may have contributed to variance in behavior, a number of exploratory analyses were performed. For this purpose, analyses focused on the percent change between active and sham rTMS, using a combined performance measure by averaging performance across the two tasks, and concatenating results from both Cohorts in the hard difficulty condition (see Section 3.3). This metric was correlated with stimulation amplitude, expressed as a percentage of the maximum stimulator output (MSO) and of the resting motor threshold (rMT), fMRI activation expressed as the *z*‐score at the stimulated ROIs in the DRAT versus DRMT contrast, and modal controllability at the stimulated ROI. Since two targets were stimulated, DLPFC and LPC, the rTMS effect was examined separately for these two targets, even though we did not find any significant differences between them.

Given the importance of the dorsal attention network (DAN) in WM, a final exploratory analysis investigated whether subjects recruiting greater activity in the DAN compared to the default mode network (DMN) benefited more from rTMS. To answer this question, a group independent component analysis (ICA) of the four runs of functional acquisitions was conducted with the GIFT toolbox (https://trendscenter.org/software/gift/). ICA was applied to extract 20 components using the InfoMax algorithm. Across the 29 completers in the rTMS analysis, four participants were excluded as they did not complete all four blocks of functional acquisitions, or due to noisy signal. The ICA was therefore performed on 25 participants. To identify the DAN and the DMN, a spatial cross‐correlation was performed with the seven‐networks parcellation from Yeo et al. (2011). The IC components showing the strongest correlation values with the DMN, and DAN defined by the Yeo atlas (Yeo et al., 2011) were selected. A cross‐correlation was also performed between the IC components and the group activation map (DRAT versus DRMT contrast), to identify the task‐component. The task component and the DAN were found, as expected, to be the same IC component corresponding to the DAN. The functional connectivity between the DMN and the DAN was then assessed and correlated with the rTMS effect.

## 3 RESULTS

The results below are organized into four sections. The first section provides results from planned analyses with participants from Cohort 1 who performed the task with one difficulty level. The second section presents results from Cohort 2, who performed the tasks with two difficulty levels. The third section presents the collapsed results from both cohorts using only the hardest difficulty level, as well as information about blinding quality across the two cohorts, and a figure presenting the stimulation target for each individual on the same brain. The last section presents exploratory analyses to test potential covariates that may capture variance in the difference between active and sham behavior.

### 3.1 rTMS effects on the Hard difficulty level (Cohort 1)

ANOVA performed on accuracy data for fifteen participants who performed the task with just the Hard difficulty level revealed a significant main effect of Task (*F*(1,14) = 104.65, *η* ^2^ = 0.68, *p* < 0.01) with better accuracy on the DRMT (92.27 ± 5.49%) than on the DRAT (69.84 ± 10.36%), as would be expected based on the added cognitive operation required to alphabetize the letters. There were no significant main effects (Target: *F*(1,14) = 2.04, *p* = 0.18; Stimulation: *F*(1,14) = 0.15, *p* = 0.70) or interactions for any of the other factors.

### 3.2 rTMS effects on Hard and Easy difficulty levels (Cohort 2)

rANOVA performed on accuracy data for the fourteen participants who performed the task with two difficulty levels revealed a significant main effect of Task (*F*(1,13) = 46.60, *η^2^* = 0.13, *p* < 0.01), with higher accuracy on the DRMT than on the DRAT (87.43 ± 7.17% vs. 78.47 ± 7.50%). A significant main effect of Difficulty was also found (*F*(1,13) = 135.93, *η^2^* = 0.28, *p* < 0.01) with higher accuracy for Easy (89.37 ± 6.59%) than Hard trials (76.46 ± 7.83%). The main effect of Stimulation was not significant (*F*(1,13) = 3.49, *p* = 0.09; active: 84.01 ± 6.97% vs. sham 81.88 ± 7.67%). The effect of Target was not significant (*F*(1,13) = 0.67, *p* = 0.43).

A significant interaction was found between Task and Difficulty (*F*(1,13) = 36.61, *η^2^* = 0.05, p < 0.01) and Bonferroni‐corrected post hoc *t*‐tests showed significant differences between the Easy and Hard difficulty levels for DRAT (Easy: 87.07 ± 7.63% vs. Hard: 68.35 ± 7.84%; *t* = 12.84, p < 0.01), and for the DRMT (Easy: 91.86 ± 6.84% vs. Hard: 83.87 ± 9.17%; *t* = 5.12, p < 0.01); a significant difference between the DRAT and DRMT at the Hard difficulty level (*t* = −9.06, *p* < 0.01), but not at the Easy difficulty level (*t* = −2.11, *p* = 0.28). A three‐way interaction was found between Task, Difficulty, and Stimulation (*F*(1,13) = 5.54, *p* = 0.04), but none of the contrasts between active and sham rTMS were significant after Bonferroni correction (Table 2).

**TABLE 2:** Mean accuracy and standard deviation (in percentage) for the three‐way interaction between Task (DRAT and DRMT), Difficulty (Easy and Hard), and Stimulation (Active and Sham)

Task:		DRAT		DRMT	
Difficulty:		Easy	Hard	Easy	Hard
**Stimulation**:	Active rTMS:	88.13 ± 6.95	70.54 ± 9.42	93.41 ± 5.84	83.93 ± 8.09
	Sham rTMS:	87.14 ± 8.90	67.54 ± 7.66	89.06 ± 9.59	83.27 ± 10.88
Bonferroni‐corrected *p*‐values		1.00	1.00	0.51	1.00

### 3.3 Combined rTMS effects on the Hard difficulty level (all participants)

**FIGURE 3:**
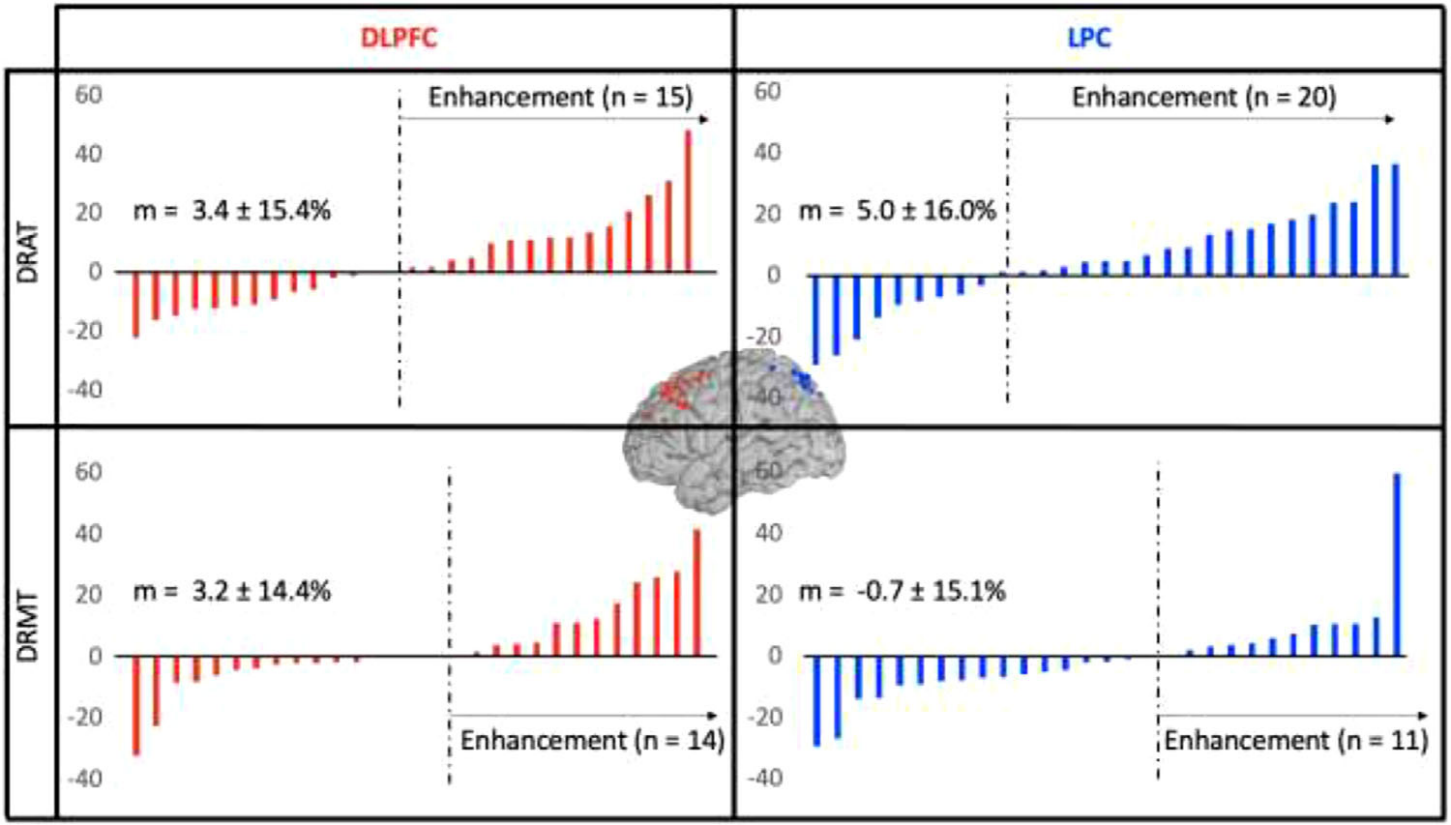
rTMS effect (expressed as a percentage of change in accuracy between active and sham rTMS) for each subject and each condition

Since the task design varies between the two cohorts by having easy trials intermixed among other trials that could impact performance via priming or next‐trial effect, we first conducted two‐sample *t*‐tests to ensure there were no significant differences in the overall accuracy (collapsed across all other factors) and reaction times. No significant differences were found between both cohorts (*Cohort 1*: 81.3 ± 7.27% vs. *Cohort 2*: 76.7% ± 7.30%, *t*_27_ = 1.63, *p* = 0.11). Similarly, there were no significant difference in accuracy when calculated as the absolute set size, rather than the relative difficulty level (set size 5 = 75.7% (*n* = 4), set size 6 = 80.4% (*n* = 17), set size 7 = 79.8% (*n* = 7), set size 8 = 79.7% (*n* = 1); *F*(3,25) < 1). The reaction times of correct trials at the hardest difficulty level did not reveal any differences between cohorts (Cohort 1: 1709 ± 228 ms vs. Cohort 2: 1771 ± 258 ms, *t*_27_ = −0.69, *p* = 0.49). Therefore, this suggests that behavioral performance between the two modified designs in the two cohorts was comparable. As such, the full sample of 29 subjects, all tested on the Hard difficulty level, were analyzed together. Results for accuracy revealed a main effect of Task (*F*(1,28) = 143.32, *η^2^* = 0.56, *p* < 0.01), with significantly lower accuracy on the DRAT (69.49 ± 9.16%) than on the DRMT (88.11 ± 8.41%). No other factors led to significant main effects (Target: *F*(1,28) = 0.33, *p* = 0.57; Stimulation: *F*(1,28) = 1.65, *p* = 0.21) was found. No significant interactions were found between Stimulation and any other factor (Task × Stimulation: *F*(1,28) = 1.44, *p* = 0.24; Target × Stimulation: *F*(1,28) = 0.23, *p* = 0.64; Task × Target × Stimulation: *F*(1,28) = 1.01, *p* = 0.32). rTMS effect expressed as a percentage of change in accuracy between active and sham rTMS, for each participant and each condition, are provided in Figure 3. An analysis of reaction times was also performed, even though this measure was not emphasized in this study. The repeated measure ANOVA revealed a main effect of Task with faster reaction times for DRMT (1652 ± 250.60 ms) than for DRAT (1854.49 ± 331.05 ms) (*F*(1,28) = 11.61, *p* = 0.002, *η^2^* = 0.14). As expected, from our previous studies using these tasks, no effects of Target (*F*(1,28) = 0.41, *p* = 0.53), Stimulation (*F*(1,28) = 0.73, *p* = 0.40)) or interaction between these factors were found.

**FIGURE 4:**
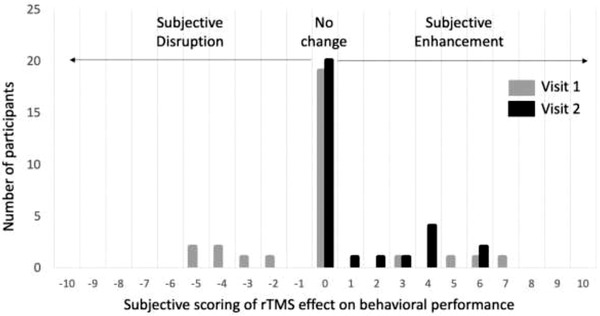
Histograms representing the subjective changes in behavioral performance associated with rTMS for each visit. Negative scores indicate performance disruption, positive scores indicates performance enhancement, and null scores indicate no changes associated with rTMS

In order to estimate the sensitivity of the observed effects and offer proscriptions for future studies, we estimated Cohen's d values for the effect of rTMS for each task and stimulus location. We observed effects sizes of 0.12, 0.24, 0.14, and −0.16 for the DRAT‐Frontal, DRAT‐Parietal, DRMT‐frontal, and DRMT‐parietal rTMS conditions, respectively. This limited effect size is likely due to differences in the relative task difficulty, as we have previously established larger and more reliable effects in previous work using similar task paradigms with a broader range of difficulties (Beynel, Davis et al., 2019). Thus, future studies might want to use harder‐to‐perform tasks, taxing the neural systems which they propose to modulate with exogenous stimulation. Regarding the quality of the blinding, participants were asked whether they thought rTMS had affected their performance on a scale from −10 to +10 with negative scores indicating disruption, positive scores indicating enhancement and 0 indicating no effects. Out of the 29 completers, only one reported a block‐to‐block difference on the first visit moving from −5 with sham rTMS to −2 with active rTMS, suggesting that they did not notice any obvious differences that would impact their behavioral performance. However, the large majority of participants did not report any rTMS effects on behavioral performance (see Figure 4)

### 3.4 Exploratory analyses

**FIGURE 5:**
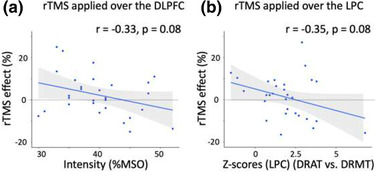
Scatterplots between: (a) Stimulation amplitude (%MSO) and rTMS effect (the percentage of change in accuracy between active and sham rTMS) when stimulating the DLPFC and (b) *Z*‐scores from the DRAT versus DRMT contrast in the stimulated ROI within LPC and rTMS effect obtained when stimulating the LPC

When correlating the rTMS effect (expressed as a percentage of change in accuracy between active and sham rTMS in the Hard difficulty level across both tasks) obtained when stimulating the DLPFC, weak correlations were found with modal controllability (*r* = 0.29, *p* = 0.13), or *z*‐scores at the stimulated site (*r* = 0.13, *p* = 0.52). However, a near significant correlation was found between rTMS effect and stimulation amplitude expressed as a percentage of MSO (*r* = −0.33, *p* = 0.08, Figure 5a), suggesting that subjects receiving lowest stimulation amplitude were the ones benefiting the most from rTMS. Interestingly, this interaction trend did not exist when the amplitude was expressed as a percentage of rMT (*r* = −0.17, *p* = 0.38).

Comparison of the rTMS effect obtained when stimulating the LPC and the other covariates revealed a negative relationship between behavioral difference and the *z*‐score of the stimulated ROI (*r* = −0.34, *p* = 0.08, Figure 5b). While this relationship did not reach statistical significance either, this pattern suggests that individuals showing less activation in the DRAT compared to the DRMT may be benefiting more from rTMS. This result is consistent with our previous findings (Beynel et al., 2020). For the other three covariates: stimulation amplitude (expressed in %MSO, and in %rMT), and modal controllability, no correlations were found with the rTMS effect (modal controllability: *r* = −0.21, *p* = 0.28; amplitude (%rMT): *r* = 0.01, *p* = 0.97; amplitude (%MSO): *r* = 0.08, *p* = 0.69).

As a further contrast of potential interest, functional DAN/DMN connectivity was correlated with the rTMS effect. While negatively related, the correlation did not reach statistical significance (*r* =−0.22, p = 0.28).

## 4 DISCUSSION

In this study, online 5Hz rTMS was applied over the left DLPFC and the left LPC as participants performed an individualized within‐subject design involving maintenance (DRMT) and manipulation (DRAT) of information held in working memory. Based on our previous studies using variants of these tasks and stimulation parameters (Beynel, Davis et al., 2020; Beynel, Davis et al., 2019), a double dissociation was expected between the stimulation sites and tasks, with active rTMS expected to impair behavioral performance of WM maintenance when applied over LPC but to improve performance when applied over the DLPFC. Conversely, active rTMS was expected to impair behavioral performance with WM manipulation when applied over the DLPFC and improve performance when applied to the LPC. While the current study produced robust main effects of task and difficulty level, illustrating the validity of the behavioral experiment, the expected effects of active and sham stimulation were not replicated. Such a failure‐to‐replicate fits with recent meta‐analytical findings demonstrating that online rTMS frequently does not modulate cognitive performance (Beynel, Appelbaum et al., 2019) and may stem from a number of design considerations or limitations of rTMS for this purpose. The following sections discuss these possibilities as they relate to the task, stimulation parameters and targeting approaches.

### 4.1 Task difficulty

In order to test the specificity of TMS on different working memory operations, the present design sought to build on our previous studies while striking a balance between the presence of multiple factors‐of‐interest and the need to collect enough trials per condition to obtain sufficient signal‐to‐noise for statistical resolution. In an attempt to accomplish this, a number of task parameters were modified relative to the previous designs. For example, “New” trials—trials where the probe letter was not in the original memory array—were excluded from this design. Moreover, because the previous studies demonstrated that significant effects were limited to the hardest conditions in the WM task, the current study was designed to focus on these harder trials and excluded the easier trials. For the first 15 participants, dubbed Cohort 1, only a single Hard difficulty level, defined by the sigmoidal fit of the visit 1 data, was used. Through an intermediate analysis of the data at the halfway point of the study, it was discovered that accuracy on the Hard condition had elevated from approximately 56% in the previous studies, to about 70% in this study. We theorized that this change may have resulted from a reduction in task variability, and therefore added the Easy condition back into the design for the remaining subjects, referred to as Cohort 2. This change, however, did not cause Hard trial accuracy to decrease to levels observed in the previous studies, but rather it remained at 68%. It can now, therefore, be inferred that additional cognitive costs due to task switching in the presence of the New trials may have been necessary to maintain such high difficulty. The absence of this factor in the current study may have contributed to the lack of active versus sham differences, and future studies may wish to include some element of task switching in an attempt to better replicate the previous designs.

### 4.2 Stimulation amplitude

Stimulation amplitude is widely regarded as an important determinant of rTMS effects (Siebner & Rothwell, 2003). The TMS amplitude, together with the coil placement and the individual head anatomy, determines the E‐field strength in the brain, which, in turn, mediates the neuromodulation at the target (Peterchev et al., 2012). Despite this, the majority of studies rely on relatively imprecise approaches for standardizing the induced E‐field that is delivered to the desired cortical target. For example, delivering stimulation at a fixed percentage of resting motor threshold is useful for normalizing intensity across individuals according to their induced motor response, but it does not properly account for differences in cortical geometry or physiology, which vary considerably across targets and between individuals.

In the current study, stimulation amplitude was defined using E‐field modeling in an attempt to ensure that all subjects received the same level of E‐field dose in the stimulated target. This desired intensity was set to 56 V/m, which had been calculated as the 100th largest E‐field magnitude (E_100_) within a parietal ROI and averaged over a sample of nine subjects from our former study, in which TMS was applied over the parietal cortex at their respective rMTs. This procedure, which has been tested and found to be effective in (Beynel, Davis et al., 2020), led to stimulation intensities that were below resting motor threshold for the majority of the current participants. Indeed, as illustrated in Table 2, out of 29 participants, only five for rTMS over the DLPFC and thirteen for LPC stimulation, received stimulation above their calculated rMT intensity. Since no significant correlation was found between the rTMS effect and stimulation amplitude expressed as a percentage of rMT, the lack of superiority of active rTMS over sham cannot be attributed to this aspect of dose individualization. Further, a recent fMRI study demonstrated that, contrary to suprathreshold stimulation which induces robust BOLD activation, subthreshold stimulation of the DLPFC is insufficient to induce significant activation, and is associated with more intersubject variability (Tik et al., 2019). Therefore, it is possible that our stimulation intensity, even if individualized, might not be ideal to induce significant changes at the behavioral level. It should also be noted that the lower average rTMS amplitude relative to both rMT and MSO in DLPFC versus LPC is expected, since the scalp‐to‐cortex distance is shorter in DLPFC and hence lower coil current amplitudes are needed to deliver the same E‐field strength to the cortical target.

When expressed as a percentage of the MSO, a trending negative relationship was observed between the stimulation amplitude and magnitude of behavioral differences for active versus sham stimulation, suggesting that lower intensities may lead to greater behavioral rTMS effects. Since scalp and auditory stimulation by TMS are directly related to the coil intensity setting as percentage of MSO, higher intensities may have caused more distraction. Finally, the average stimulation amplitude for rTMS applied over LPC in this study and in our former study (Beynel, Davis et al., 2020), showing a superiority of active rTMS, did not differ significantly (47.72 ± 10.43% MSO vs. 46.93 ± 8.24% MSO), suggesting that the lack of significant differences between active and sham rTMS is not related to the stimulation amplitude.

### 4.3 Targeting approach

Based on previous observations that task‐specific fMRI BOLD activation and modal controllability correlated with rTMS behavioral effect (Beynel, Deng et al., 2020), these two factors were used in the current study to define the DLPFC and LPC stimulation targets. To our knowledge, this study is the first to combine these two neuroimaging approaches. It has been demonstrated that using an individualized fMRI targeting approach significantly enhances rTMS efficacy, both in a study directly comparing different targeting approaches (Sack et al., 2009) and in a large meta‐analysis comparing targeting approaches across more than one hundred studies (Beynel, Appelbaum et al., 2019). On the other hand, modal controllability, the ability of one node to bring the whole brain into a hard‐to‐reach states has never been used as a targeting approach. According to our recent results (Beynel, Deng et al., 2020) and the ones from others (Medaglia et al., 2018) suggested that considering structural network dynamics might improve rTMS efficacy.

However, contrary to expectations this targeting approach did not improve the effect size compared to our former study. No significant correlation was found between rTMS effect and modal controllability, and only a trending negative relationship was found between rTMS effect and fMRI BOLD activation. Taken together this could suggest that rTMS efficacy does not depend on these approaches. Nevertheless, no strong conclusions can be drawn from this result given the lack of a significant rTMS effec.

In this study, a large number of factors that were included and randomized to investigate the specificity of rTMS effect. However, this also prevented testing of certain specific effects and interactions. For example, the effect of learning could not be investigated since it was confounded with the effect of stimulation site. While the results demonstrated a main effect of time, when collapsed across all other factors (*t*_28_ = −2.88, *p* < 0.01), it could not be attributed to practice alone. Future studies might include a more complete randomization to untangle these factors.

To increase the sample size, the results of the two cohorts were collapsed. Even though the results did not reveal any differences in the reaction times or accuracy levels between them, there are important differences since easier trials are intermixed with harder trials in the second cohort. It is well known that performance to a trial is highly influenced by performance to the former trial and therefore including a full cohort performing the exact same task would probably have helped strengthening the results.

Regarding rTMS parameters, we opted for online rTMS application, since it has been proposed that applying rTMS while activating the stimulated network might potentially produce stronger effects through Hebbian‐like plasticity (Sathappan et al., 2019). However, this approach requires finding the proper temporal synchronization between the TMS trains and the ongoing brain activity. One study suggested that rTMS effects are transient and are unlikely to produce behavioral effects that last beyond the stimulation train (Hamidi et al., 2011). Our previous work using this same paradigm found no significant effect of Stimulation timing (i.e., rTMS applied immediately before the encoding of the letter array, or during the short delay period) on response accuracy or reaction time (Beynel et al., 2019), and we were therefore confident that there would be no difference in TMS effect for the current study based on similar timing decisions. Nonetheless, it is possible that applying rTMS right before the encoding might not be the ideal timing to observe behavioral effects, even though our previous studies did not demonstrate any significant differences on behavioral performance with rTMS was applied before the encoding or during the manipulation of the letters. Interestingly, a recent study applying rTMS over the DLPFC during (i) the maintenance period of a working memory task (ii) the rest period of the working memory task, or (iii) without any cognitive task (Bakulin et al., 2020), demonstrated that the effect of rTMS on a behavioral task performed after stimulation, were found only when stimulation was applied when subjects were at rest, suggesting that the Hebbian‐like plasticity of rTMS remains to be understood.

### 4.4 Conclusions

While the current study was well‐motivated based on past findings, the observed patterns of results here did not reveal the expected double disassociation between target location and active‐versus‐sham behavioral effects. Though the current study attempted to strike a balance between the many task and stimulation factors while implementing advanced targeting and computational modeling approaches, it appears that the task used here may have been overly sensitive to subtle experimental parameters changes that may have mitigated robust TMS effects. Future studies may wish to focus on a psychometrically stable WM measure, rather than prioritizing individualized procedures, as done in this study. Results from this study also demonstrates that defining individualized targets with fMRI data is challenging, even with well‐characterized domains like working memory; as such, relying on statistical techniques developed for group analyses may not provide the most flexible technique for identifying subject‐level functional target. Furthermore, use of closed‐loop approaches with EEG may also yield greater efficacy of online techniques, such as those used in this study, and such developments are strongly encouraged.

## CONFLICT OF INTERESTS

Angel V. Peterchev is an inventor on patents and patent applications on TMS technology. Related to TMS technology, Angel V. Peterchev has received research and travel support as well as patent royalties from Rogue Research; research and travel support, consulting fees, as well as equipment donation from Tal Medical / Neurex; patent application and research support from Magstim; equipment loans and hardware donations from MagVenture; and consulting compensation from Neuronetics, BTL Industries, and Advise Connect Inspire.

### PEER REVIEW

The peer review history for this article is available at https://publons.com/publon/10.1002/brb3.2361

## Data Availability

Data from this manuscript are available on OSF: https://osf.io/yj8em/
